# Vitamin B12 and D3 Levels in Leprosy Patients With or Without Deformity: A Cross-Sectional, Analytic Study

**DOI:** 10.7759/cureus.85376

**Published:** 2025-06-04

**Authors:** Divya Saini, Ghazal Ahmed, Habib Md R Karim

**Affiliations:** 1 Medicine, All India Institute of Medical Sciences, Deoghar, IND; 2 Dermatology, Venereology, and Leprosy, All India Institute of Medical Sciences, Deoghar, IND; 3 Anesthesiology, Critical Care, and Pain Medicine, All India Institute of Medical Sciences, Guwahati, IND

**Keywords:** hansen’s disease, limb deformity, nutrition and dermatology, vitamin b 12, vitamin d serum level

## Abstract

Background: Considering the role of vitamins B12 and D3 in musculoskeletal, bone, and nerve health, their possible role in deformity among leprosy patients remains to be investigated. The study compared vitamin B12 and D3 levels in leprosy patients with and without deformity, aiming to identify the cut-off points that predict deformity and neuritis.

Methods: With approval and informed consent, clinic-demographic, leprosy disease, and deformity-related data, as well as vitamin B12 and D3 levels, were recorded among both new and established leprosy patients attending the outpatient department from mid-February to mid-May 2024 and were categorized based on the presence or absence of deformities for comparison. Areas under the receiver operating characteristic curves (AuROC) and Youden’s J point for vitamin B12 and D3 levels, predicting deformity and neuritis, were also evaluated. Statistical significance was set at p < 0.05.

Results: Data from 50 participants (21 with deformities) were analyzed. Patients with deformities were less educated compared to those without (p = 0.03). Similarly, the number of homemakers, daily wagers, and farmers was significantly higher in the deformities group. The prevalence of B12 and D3 deficits was 2 (9.52%) and 4 (19.04%) in patients with deformity and three (10.34%) and one (3.45%) in patients without deformity, respectively. Neuritis was more common in the deformity group (42.86% vs. 17.24%); however, the levels of B12 and D3 were statistically equivalent. AuROC (95% confidence) for B12 level and D3 for deformity were 0.405 (0.237-0.573) and 0.599 (0.434-0.764), and for neuritis, 0.645 (0.464-0.826) and 0.508 (0.339-0.677), respectively.

Conclusion: The present study showed an insignificant relationship between Vitamin B12 and Vitamin D3 levels in patients with and without deformities. Similar results were observed in neuritis. However, a relationship was found between socioeconomic status and deformity in patients with leprosy. Our interpretation, however, is limited by a single-center, nonlongitudinal design of study with a small sample size and a single testing occasion.

## Introduction

Leprosy remains a significant public health concern in India, with certain regions showing particularly high prevalence. Multiple studies have examined the nutritional status of patients, focusing on the role of macro- and micronutrients, as well as their receptors, in the pathogenesis and severity of leprosy. However, a recent systematic review was inconclusive regarding the association between nutritional factors and disability in leprosy [1]. Even data on deformities is limited. Nutrients are crucial in maintaining musculoskeletal and neurological function, with vitamins D3 and B12 being essential [2,3]. Low levels of vitamin B12 have been associated with impaired peripheral nerve function, both sensory and motor, suggesting that such deficiencies may contribute to neuromuscular impairment and deformities in leprosy [3].

Leprosy-related deformities vary from nail changes to more severe conditions such as claw hand, wrist drop, and foot drop [4]. These deformities not only result in physical disability but also have significant psychological and social implications. Epidemiological studies suggest that around one-third of leprosy patients experience deformities [4]. A 20-year retrospective analysis from a hospital outpatient department in North India reported a deformity rate of 66% in cases of pure neuritic leprosy [5]. Given the high burden of deformities, identifying a potential link with nutritional deficiencies could support preventive strategies. Nonetheless, despite extensive research, recent systematic reviews have not established definitive predictors of deformity [1]. This study aimed to compare serum vitamin B12 and D3 levels in leprosy patients with and without deformities. The secondary objectives were to determine the prevalence of vitamin B12 and D3 deficiencies among leprosy patients and to identify cut-off values predictive of deformity.

## Materials and methods

Study design and settings

This was a prospective, cross-sectional, analytic, parallel-arm study, categorizing the cohort into case and control, conducted in the Dermatology, Venereology, and Leprosy Outpatient Department (OPD) of the All India Institute of Medical Sciences, Deoghar, India. The institutional research board cleared the project, which was subsequently approved by the institutional ethics committee (No. AIIMS/DEO/RC-IEC-Subcommittee/2023-Sept/28). The study was conducted from January to June 2024, with data collection taking place from mid-February to mid-May of the same year.

Sample size

This study was part of the Indian Council of Medical Research Short-Term Studentship Research Project (Reference ID: 2023-10185). Similar studies and data were not found in our preliminary literature search to base our sample. Therefore, we planned the study using conventional sampling to recruit all consenting participants over a period of two months.

Participants

All leprosy patients, regardless of whether they were new or existing cases in the adult age group, including both males and females with or without deformities, were approached for recruitment. Patients who consented to participate were enrolled in this study. Patients who had already received vitamin B12 or vitamin D supplements were also excluded from the study. Group allocation will be based on the presence of deformity at the time of diagnosis. Group A consisted of leprosy patients with deformity, while Group B consisted of patients without deformity.

Data collection and outcome variables

Age, sex, weight, height, and derived demographic data were collected. A clinical examination-based interview was conducted to gather information pertinent to the pre-approved case record form. On the day of group allocation, blood samples were taken from both groups to assess vitamin B12 and D3 levels. B12 and D3 levels were evaluated using a chemiluminescent immunoassay with the VITROS 5600 H analyzer system (Ortho Clinical Diagnostics, now known as QuidelOrtho, San Diego, United States). Furthermore, data on disease-related illness duration, family background, economic status, profession, and dietary habits were collected. Deformities were also noted in the group with deformities.

Data management and statistical analysis

MS Excel (Microsoft Corporation, Redmond, Washington, United States) was used to prepare the master chart. Categorical variables are presented as absolute numbers and percentage scales, and continuous data are presented as mean ± standard deviation. Data distribution was assessed using the k-test, and groups were compared using the unpaired t-test or the Mann-Whitney test. Contingency table data were compared using Fisher's exact test. Statistical significance was set at p < 0.05. INSTANT software (GraphPad Prism, La Jolla, California, United States) was used for statistics. AuROC and Youden’s J point analysis were performed online using Epitools Epidemiological Calculators.

## Results

A total of 57 patients were screened. Two patients who were already on vitamin B12 and D3 were excluded. Fifty-five participants were recruited during the study period. Data from five patients were grossly deficient and excluded from the analysis. The B12 levels of two patients were unavailable; therefore, they were replaced with the group's mean values. Twenty-one (42.0%) patients had deformities, and twenty-nine (58.0%) did not. Although patients with deformity were older and had a preponderance of male sex, the differences were not statistically significant. Patients with deformities were low-educated (illiterate or primary-level education) compared to patients without deformities (p-value: 0.03). Similarly, the number of homemakers, daily wagers, and farmers was significantly higher in the deformities group (Table 1).

**Table 1:** Clinicodemographic details of the leprosy cohort with and without deformity compared using Fisher's exact test and #unpaired t-test SD: standard deviation

Parameters	All (N = 50), n (%)	With deformity (N = 21), n (%)	Without deformity (N = 29), n (%)	p-value
Age (years) mean ± SD	39.22 ± 15.7	41.24 ± 17.1	37.76 ± 14.78	0.44^#^
Male	30 (60.0)	15 (71.43)	15 (51.7)	0.24
Female	20 (40.0)	6 (28.57)	14 (48.3)	
Illiterate	13 (26.0)	5 (23.81)	8 (27.59)	0.01
Primary	18 (36.0)	12 (57.14)	6 (20.69)	
High school	16 (32.0)	2 (9.5)	14 (48.27)	
Secondary and above	3 (6.0)	2 (9.5)	1 (3.45)	
Illiterate and primary	31 (62.0)	17 (80.95)	14 (48.28)	0.03
High school and above	19 (38.0)	4 (19.05)	15 (51.72)	
Homemaker	16 (32.0)	4 (19.05)	12 (41.39)	0.03
Daily wager/laborer/vendor	14 (28.0)	9 (42.85)	5 (17.24)	
Farmer	7 (14.0)	5 (23.81)	2 (6.89)	
Student/unemployed	9 (18.0)	3 (14.29)	6 (20.69)	
Salaried (private/public)	4 (8.0)	0	4 (13.79)	
Disease duration (months) mean ± SD	25.8 ± 52.3	24.7 ± 52.0	26.6 ± 53.5	0.90^#^
Tuberculoid	1 (2.0)	0	1 (3.45)	0.04
Borderline tuberculoid	20 (40.0)	5 (23.81)	15 (51.72)	
Borderline	3 (6.0)	0	3 (10.35)	
Borderline lepromatous	9 (18.0)	5 (23.81)	4 (13.79)	
Lepromatous	17 (34.0)	11 (52.38)	6 (20.69)	
Lepra reaction-total	31 (62.0)	13 (61.9)	18 (62.07)	0.15
Lepra reaction-type 1	4 (8.0)	0	4 (13.79)	
Lepra reaction-type 2	15 (30.0)	8 (38.1)	7 (24.14)	
Neuritis n(%)	14 (28.0)	9 (42.86)	5 (17.24)	0.06
Hemoglobin level (gm/dL)	11.9 ± 1.6	11.6 ± 1.9	12.2 ± 1.3	0.22^#^

**Figure 1:**
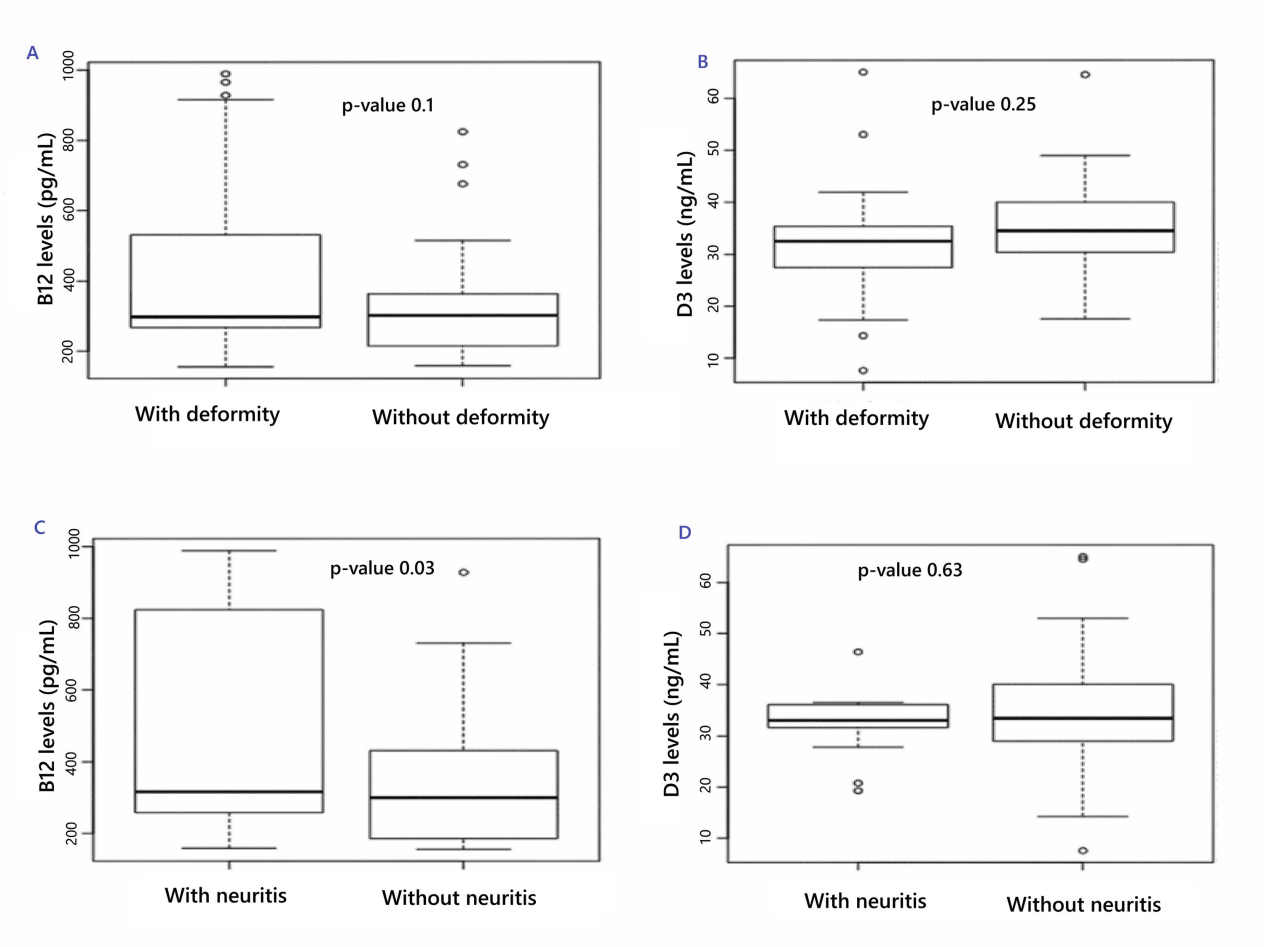
Box and Whisker plot comparison of (A) B12 level and (B) D3 levels with and without deformity and (C) B12 level and (D) D3 levels with and without neuritis tested using unpaired t-test

The prevalence of B12 and D3 deficits was 2 (9.52%) and 4 (19.04%) in patients with deformity and 3 (10.34%) and 1 (3.45%) in nondeformity patients, respectively. The incidence of neuritis was 42.86% in the group with deformity and 17.24% in the group without deformity. However, B12 and D3 levels were statistically indifferent to deformities (Figure 1). The result was similar for D3 in the case of neuritis. Interestingly, B12 levels were higher in patients with neuritis. The statistical central tendencies and their dispersion are listed in Table 2.

**Table 2:** Statistical central tendencies and their dispersions for vitamin B12 and D3 in leprosy patients with and without deformity tested using unpaired t-test SD: standard deviation

Agent	Outcome	Min	5%	25%	Median	75%	95%	Max	Mean	SD	p-value
Vitamin B12 (pg/mL)	With deformity	156	159	268	298	531	965	989	437	281	0.1
	Without deformity	159	159	215	302	364	709	824	330	176	
Vitamin D3 (ng/mL)	With deformity	7.6	14.3	27.4	32.5	35.4	53	65	31.6	12.6	0.25
	Without deformity	17.5	21	30.4	34.5	40	48	64.5	35.2	9.47	
Vitamin B12 (pg/mL)	With neuritis	159	206	260	316	752	973	989	486	306	0.03
	Without neuritis	156	159	188	300	427	690	928	332	179	
Vitamin D3 (ng/mL)	With neuritis	19.3	20.3	31.6	33	35.9	40	46.4	32.5	6.72	0.63
	Without neuritis	7.6	16.6	29	33.5	40	55.9	65	34.2	12.2	

**Figure 2:**
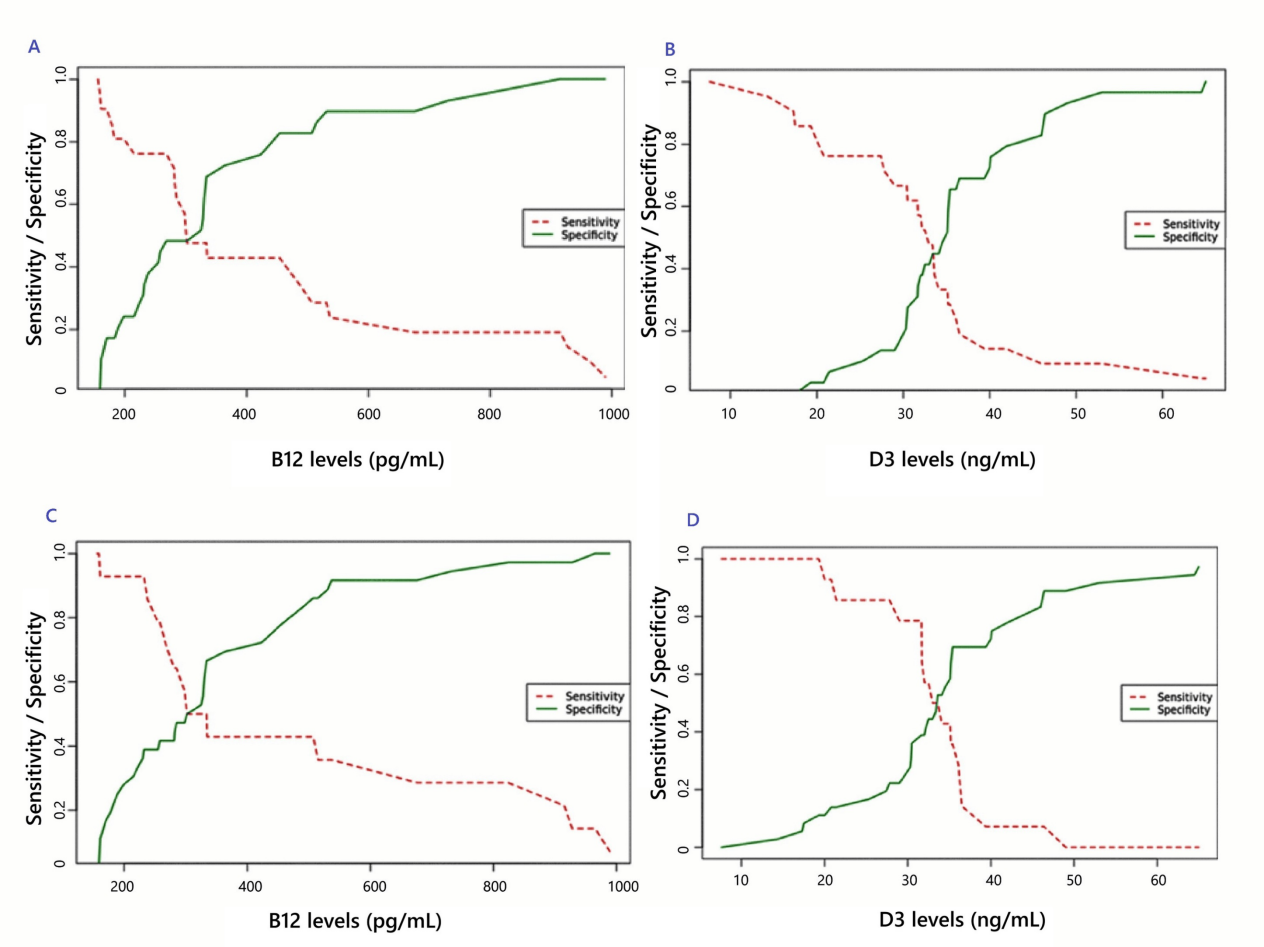
Two-curve receiver operating characteristic for (A) B12 with deformity, (B) D3 deformity, (C) B12 with neuritis, and (D) D3 with neuritis in leprosy patients

The area under the receiver operating characteristic curve (AUROC) with 95% confidence for B12 level and D3 for deformity were 0.405 (0.237-0.573) and 0.599 (0.434-0.764), respectively, and for neuritis, 0.645 (0.464-0.826) and 0.508 (0.339-0.677) (Figure 2).

The box and whisker presentation and two-curve ROC for B12 with deformity, D3 deformity, B12 with neuritis, and D3 with Neuritis are presented in Figures 1-2. Youden’s J for B12 with deformity, D3 deformity, B12 with neuritis, and D3 with neuritis, along with their respective sensitivities and specificities, are presented in Table 3.

**Table 3:** Shows the Youden’s J for B12 with deformity, D3 deformity, B12 with neuritis, and D3 with neuritis, along with their respective sensitivities and specificities

Description	Youden’s J	Sensitivity	Specificity	Sensitivity + specificity
B12 (pg/mL) for deformity	454.3	0.429	0.828	1.257
D3 (ng/mL) for deformity	53	0.095	0.966	1.061
B12 (pg/mL) for neuritis	232	0.929	0.389	1.318
D3 (ng/mL) for neuritis	31.65	0.786	0.389	1.175

## Discussion

This study compared serum levels of vitamins B12 and D3 in patients with leprosy, with and without deformity at diagnosis, and found no significant difference between the groups. This was also true for the presence of neuritis. However, the proportion of patients with neuritis was notably higher among those with deformities (42.86%) than those without (17.24%). *Mycobacterium leprae* has a known predilection for peripheral nerves, leading to thickening and functional impairment. Given the interplay between the nervous and musculoskeletal systems, patients with neuritis are potentially at greater risk for trauma and subsequent deformity.

The proportion of male patients with deformities was higher (71.43%) than that of female patients (51.7%). While greater male vulnerability is often attributed to increased outdoor occupational exposure, the current study cohort primarily comprised Indigenous individuals, among whom women also actively engage in fieldwork and income-generating activities in addition to domestic responsibilities [6,7]. This may explain the lack of statistically significant gender differences in our findings, aside from occupation.

Most participants were in their third and fourth decades of life, consistent with previous studies, although some have reported a predominance among younger individuals aged 21-30 years [8,9]. As other epidemiological studies show, our data also reflect an association between low socioeconomic status, illiteracy, and leprosy [9,10].

Recent research has increasingly focused on the nutritional status of individuals with leprosy [11]. MRI studies have shown that vitamin B12 deficiency affects the cervical and thoracic spinal cord [12]. In contrast, vitamin D3 plays a crucial role in calcium and phosphorus metabolism, which is essential for maintaining bone and tendon health [2]. Multiple studies have reported a high prevalence of nutritional deficiencies among leprosy patients, and vitamin D receptor gene expression is reduced in nearly all forms of leprosy compared to healthy controls [13,14]. Although the clinical significance of vitamin D receptor expression remains uncertain, its potential link with disease progression and leprosy reactions has been suggested [15].

Nutritional comparisons across clinical subtypes have shown that patients with paucibacillary leprosy have significantly higher levels of vitamins A, E, C, D, and B12 than those with multibacillary disease [13]. Still, findings remain inconsistent. While Kumar et al. observed significantly lower levels of these vitamins in leprosy patients, Singh et al. reported no association between vitamin D levels and clinical manifestations of the disease [13,14].

In our study, vitamin B12 and D3 levels were not found to be associated with deformity or neuritis. Interestingly, B12 levels were relatively higher in patients with neuritis. However, such an association found might not be the true association, as we have tested the serum levels at only one point in time, and serum levels might vary during the illness. Further, diet might also play a crucial role, which we need to evaluate longitudinally. Nevertheless, a high proportion of patients presented with grade 2 disability, likely due to early nerve involvement [16]. Although severe disease is expected to yield more profound systemic effects, the specific relationship between vitamins D3 and B12 and deformity at diagnosis remains to be conclusively established.

This study is limited by its small sample size and single-center design. Dietary habits, influenced by socioeconomic status, were not fully accounted for; nonvegetarian preference does not guarantee regular or sufficient intake. Sunlight exposure, especially for farmers and daily wage workers, can also affect vitamin D3 levels. The widespread use of over-the-counter vitamins, combined with participants' low literacy levels, further hinders the accurate assessment of supplementation. Serum levels of the vitamin may vary over time, especially when the disease and deformity caused have a longer natural history. Therefore, single-point measurement may not show accurate insights. A larger, multicenter study with a longitudinal design is needed to validate these findings.

## Conclusions

This observational analytic study comparing serum vitamin B12 and D3 levels among patients with leprosy showed an insignificant relationship between vitamin B12 and vitamin D3 levels in patients with and without deformities. Similar results were also noted for neuritis, except that B12 levels were higher in patients with neuritis. There were slightly higher numbers of neuritis in patients with deformity than in those without. Nevertheless, a relationship was found between socioeconomic status and a low level of education with deformity in leprosy patients. However, our findings are limited by the study's single-center, nonlongitudinal design, which has a small sample size and a single testing occasion. Furthermore, we were unable to account for the roles of diet and sunlight exposure. 
